# Searching for Street Parking: Effects on Driver Vehicle Control, Workload, Physiology, and Glances

**DOI:** 10.3389/fpsyg.2020.574262

**Published:** 2020-10-20

**Authors:** Canmanie Teresa Ponnambalam, Birsen Donmez

**Affiliations:** Human Factors and Applied Statistics Laboratory, Department of Mechanical and Industrial Engineering, University of Toronto, Toronto, ON, Canada

**Keywords:** driver behavior, distraction, on-road study, instrumented vehicle, visual attention, traffic safety, parking

## Abstract

Urban areas that allow street parking exhibit a heightened crash risk that is often attributed to factors such as reduced road width, decreased visibility, and interruptions to traffic flow. No previous on-road studies have investigated how the demands of searching for parking affect driving performance, physiology, and visual attention allocation. We are interested in these effects on the driver and their possible influence on the safety of the environment. While simulator studies offer several benefits, the physical, mental and social pressures incurred by searching for parking in an urban streetscape cannot be emulated in a simulator. We conducted an on-road instrumented vehicle study with 28 participants driving in downtown Toronto, Canada to explore the effect of searching for street parking on drivers. During the experiment, participants drove two routes in a counterbalanced order: one route with a parking search task, and the other route as a baseline. Speed and lane position were measured via vehicle instrumentation, heart rate and galvanic skin response were measured through physiological sensors, and gaze position was collected through a head-mounted eye-tracker. Participants completed the NASA Task Load Index after each route. It was found that while searching for parking, participants drove slower and closer to the curb, and perceived higher workload. While there were no statistically significant effects in physiological measures, there was a rise in heart rate approaching statistical significance. A detailed analysis of eye-tracking data revealed a clear change in glance behavior while searching for parking, with an increase in long off-road glances (>2 s) and decrease in shorter off-road glances (<1.6 s). Some exhibited behaviors (e.g., slowing down) may be seen to compensate for the potentially negative effects of increased demands associated with parking search, while others (e.g., increase in long off-road glances) have the potential to increase crash risk. This study acts as an important first step in revealing changes in driving performance, physiology and glance behavior brought on by searching for parking in a real-world urban environment.

## 1. Introduction

The convenience and often limited availability of street parking makes it a coveted resource in many downtown areas. Hampshire and Shoup (2018) highlighted 22 studies in 15 different cities from 1927 to 2015 examining proportions of traffic cruising for parking. The latest (2005–2015) of these studies found an average of just under 40% of traffic cruising for parking across 3 cities. More recent studies found the number of vehicles cruising for parking to be 15% in Stuttgart, Germany (Hampshire and Shoup, 2018), 5–6% in San Francisco, CA, and 3–4% in Ann Arbor, MI (where cruising refers only to *excess* travel due to the search for parking) (Weinberger and Millard-Ball, 2017). Regardless of the variability in statistics, measurement methods and definitions, it is widely regarded that street parking can be difficult to find on demand (especially in urban areas during busy times of the day). At these times drivers may be forced to search for parking on or off their intended route. As presented in detail below, many studies document the heightened crash risk evident in areas that allow street parking. However, to the best of our knowledge, no studies have attempted to measure the effect on drivers of engaging in the parking search task, nor how drivers actively searching for parking while driving affect the safety of the road environment. In this paper, we present an on-road instrumented vehicle study investigating how drivers' vehicle control, workload, physiology, and glances are affected by searching for street parking.

There has been significant research on the safety effects of the presence of street parking. A review of street parking in the U.S. estimated that it was associated with 15% of crashes (Sisiopiku, 2001). Similarly, a 1971 report concluded that street parking was directly or indirectly responsible for 20% of all urban crashes in the U.S. (Highway Research Board, 1971). The report named five primary reasons for why street parking increases crash risk: increased obstacles (i.e., parked vehicles), disruption of traffic flow by cars leaving parking spaces, disruption of traffic flow by cars entering parking spaces, drivers or passengers exiting parked vehicles, and reduced sight distance of pedestrians. Decreased road width and sight restrictions due to parked vehicles have also been cited as an issue (Greibe, 2003; Box and Levinson, 2004; Cao et al., 2017). A study conducted by Edquist et al. (2012) showed that the visual complexity of an urban environment in the presence of parked cars can increase the workload of drivers and influence their driving behavior. In that study, there was little difference between an environment that did not allow street parking and one with empty bays, suggesting that the presence of parked cars was the most significant contributor to workload. Some have argued that searching for parking results in drivers slowing down to safer speeds, reducing crash severity (Lerner-lam et al., 1992; Daisa and Peers, 1997; Marshall et al., 2008), or that parked vehicles can protect pedestrians by separating moving traffic from the sidewalk (Lerner-lam et al., 1992). Despite these arguments and supposed safety benefits of street parking, crash risk appears to be elevated in areas that allow it.

While no studies were found that assessed the task of searching for parking, tasks that generally visually engage drivers have been shown to affect many measures of driving, such as lane position and lane position variability, speed and speed variability, reaction time to external events, and subjective workload (Regan et al., 2008). The tasks most commonly studied are voluntary in-vehicle tasks (e.g., mobile phone use) that often have a manual component in addition to visual. Few studies were found that examine visual secondary tasks without a manual component or that concern distractions outside of the vehicle. A recent analysis of the largest naturalistic driving study to date found that some observable type of distraction was involved in 68% of crashes and that extended glances to external objects were associated with a crash risk 7.1 times that of normal driving (Dingus et al., 2016). Visual search is the premier component of searching for parking and requires drivers to scan the environment on the side of the road to locate and confirm vacant spots in tandem with reading posted parking restrictions and road markings. The existence of parked cars provides an obstacle to getting within reading distance of roadside signage and contributes to the complexity of the road environment. While searching for parking, it is expected that drivers spend more time glancing off-road and exhibit an increased number of off-road glances, an effect that we aim to verify and quantify in this study. Regarding driving behavior, the addition of a visual task has been shown to result in reduced speeds and increased lane keeping variability (Dingus et al., 1995; Engström et al., 2005; Zhang et al., 2006). The same behavior was found when drivers drove in an environment with higher visual complexity (Edquist et al., 2012). Similar results are anticipated for speed and lane keeping variability when drivers are tasked with searching for parking. In addition, because sign reading is assumed to be a significant aspect of finding street parking, it is possible that drivers drive closer to the curb when searching to allow them to read posted parking restrictions, particularly due to the potentially small letter sizing (see: example in **Figure 4**). In order to quantify how drivers are affected while searching for parking, both vehicle control and glance behavior require investigation.

In addition to visual demand, searching for parking may increase cognitive demand and stress. Drivers searching for parking in an urban center could be further burdened with the task of navigating while searching for parking. In addition, the time drivers spend locating a parking space has been shown to be a major influencing factor in choosing a parking spot (Brooke et al., 2014). Time spent looking for parking is time removed from the driver's ultimate destination, making the search for parking a task best done as quickly as possible. Time urgency has been shown to relate to driver stress and affect driving behavior (Hennessy and Wiesenthal, 1999). In addition, as drivers search and obstruct traffic in busy areas, they are likely to find themselves under the pressure of following vehicles, who may honk or keep close distances. This social pressure can further contribute to the stress of the driver and pressure them to maintain a speed that makes the parking search more difficult. These cognitive load and stress effects can be assessed through various measures including self-reports (Hart, 2006) as well as heart rate and skin conductance, which are known to rise under increased stress and cognitive load (Healey and Picard, 2005; Mehler et al., 2012). These measures have been used in other on-road studies as quantifiers of stress levels in drivers; such a study found an increase in heart rate, indicating a rise in stress level, exhibited by drivers when parallel parking manually compared to parking with assistive technology (Reimer et al., 2016).

From existing research, it is unclear what (if any) the effects of searching for parking while driving are on drivers and, in turn, the road environment. To investigate this, we conducted an on-road instrumented vehicle study in downtown Toronto, Canada, to explore how drivers' vehicle control, perceived workload, physiology, and glance behaviors change while searching for parking in a busy urban area. As an inaugural step into investigating the parking task, we focus only on the search itself and not the task of parking the vehicle. To the best of our knowledge, no other research has investigated the effects of searching for parking at the driver level from any of the perspectives of vehicle control, perceived workload, physiology, and visual attention allocation.

## 2. Experimental Method

To study the effects of searching for parking at the driver level, an experiment must adequately simulate the parking search task in a controlled manner and allow for relevant measures to be recorded under representative driving scenarios. Despite the limitations of on-road studies in regards to experimental control, driving simulators have other limitations that render them less effective to study parking search. For example, it is hypothesized that the social pressure of blocking traffic is a contributor to the demands on the driver while searching for parking; this pressure cannot be induced in a simulator. In general, the perception and influence of risk is limited in a simulated environment. In addition, sign reading and visual scanning are key components of searching for parking, and simulators are limited in the resolution and visual detail they can provide, making them less effective in studies that focus on visual scanning (Kaptein et al., 1996). We therefore chose to conduct our study on the road in an instrumented vehicle. The study was approved by the University of Toronto Research Ethics Board (protocol number 32795).

As this experiment was the first to examine the effects of searching for parking, we preferred to focus on roads with attributes that pose the highest demands on drivers: complex visual environment, erratic traffic flow, and high occupancy of pedestrians and cyclists. The busy environment also ensures that others (i.e., drivers in following vehicles) are affected by changes in driving behavior, such as potential reductions of speed. This maintains the social pressure expected when searching for parking while driving. However, as this is a first step, we chose not to explicitly investigate this influence nor the influence of time pressure, though both are expected to play a role in the searching for parking task. A simulator study by Edquist et al. (2012), though they did not study the parking search itself, showed that areas with many empty parking bays did not create as high a visual demand on drivers as when there were many parked cars (90% of bays occupied), thus we also conducted the study on roads with a high occupancy of parked cars. There was one (within-subject) independent variable in this study with two levels: driving with a parking search task and driving with no-task (baseline). Participants completed two 15- to 20-min routes under the parking-search and baseline conditions. While both routes were selected to be similar in length and complexity, the order of the routes and the conditions were fully counterbalanced across the participants to remove the potential effects of route and order confounds. During the analysis, it was validated that route and order did not significantly affect the results. The experiment was run between July 2017 and October 2017, on Saturdays or Sundays, starting at either 10:30 a.m. or 1:30 p.m. Running experiments during the summer and only on weekends offered some level of control over the weather and traffic density as well as the number of pedestrians in the area, and ensured that there would be no road work or waste collection interruptions during the experiment.

### 2.1. Participants

Participants were recruited via posters placed around the university campus and on online forums. Due to insurance and Research Ethics Board constraints, participants were required to be between the ages of 35 and 54 and have a full driver's license for at least 3 years. Therefore, our sample represented a low-crash risk group (Cooper, 1990; McGwin and Brown, 1999). Participants could not wear glasses during the experiment as this affected the quality of data gathered by the head-mounted eye tracker. Therefore, only drivers who can legally drive without glasses (contacts were allowed) could participate in the study. Twenty-eight participants (14 male and 14 female, mean age 41.9, st. dev. of age 5.7) completed the experiment, however due to equipment malfunctions not all participants had full sets of data (discussed in more detail in the section 4). Of those that answered (23 participants), 80% reported that they drive a vehicle a few days a week or more. When asked how frequently they drive in the downtown location of the study, 38% of participants reported a few days a week or more, 44% reported a few days a month, and 17% a few days a year. Participants were compensated at CAN $15/h.

### 2.2. Apparatus

**Figure 1:**
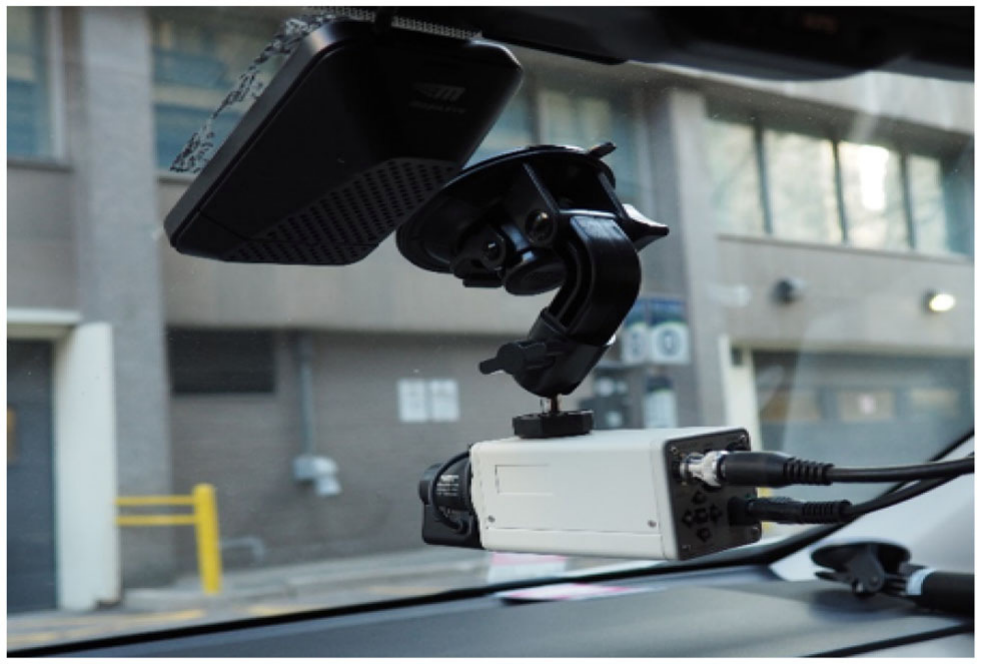
MobilEye (black unit) and front-facing camera mounted on windshield.

The instrumented vehicle was a 2014 Toyota RAV4 equipped with a MobilEye device to sample data from the Controller Area Network (CAN bus) connection. The MobilEye also provided measures calculated through image processing techniques applied to video from its internal camera (such as lane position). Another camera mounted on the dashboard provided video of the front-view of the vehicle. Both the MobilEye and front-facing camera can be seen in Figure 1.

**Figure 2:**
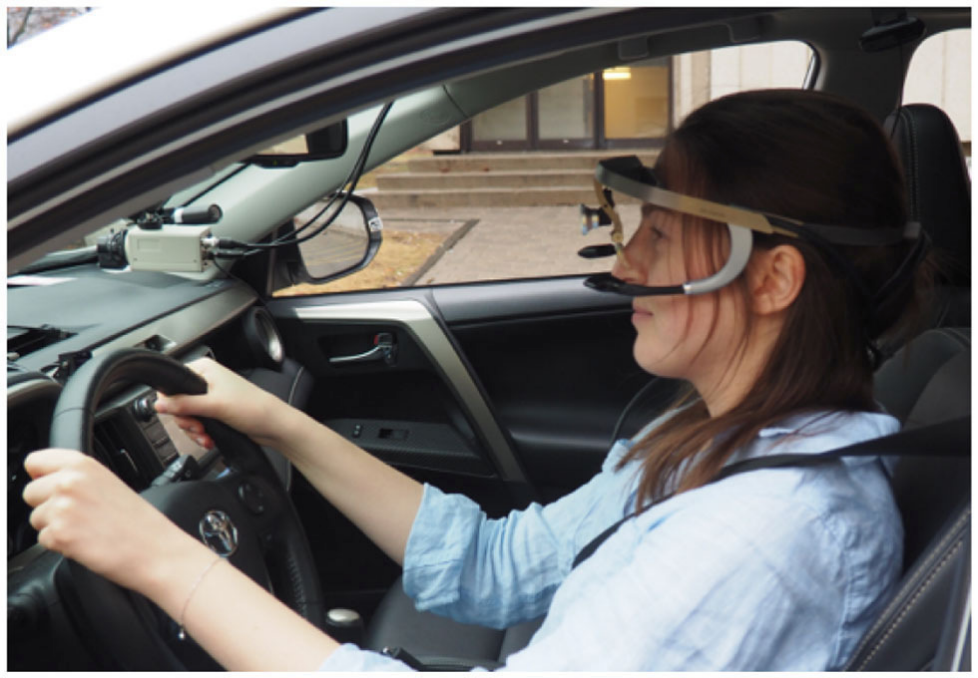
Driver outfitted with Dikablis eye-tracking glasses.

Electrocardiogram (ECG) and galvanic skin response (GSR) sensors produced by Becker Meditec were used to measure heart rate and skin conductance, and recorded data at 240 Hz; three electrodes were placed on the chest to read the ECG signal and two electrodes were placed on the bottom of the left foot to obtain the GSR signal. Both the hand and foot are popular placements for GSR measurement in driving studies; one study investigated both placements and found both to be feasible (Avcı et al., 2014). We opted for the foot placement as we presumed the wires would then be less disturbing to the driver while driving. Gaze position was captured using the head-mounted Dikablis Eye-Tracking Glasses (Figure 2), produced by Ergoneers. When calibrated, this device uses two cameras pointed toward the eyes to determine gaze position (tracked at 50 Hz) and overlays the gaze position on video data captured by its front-view camera.

**Figure 3:**
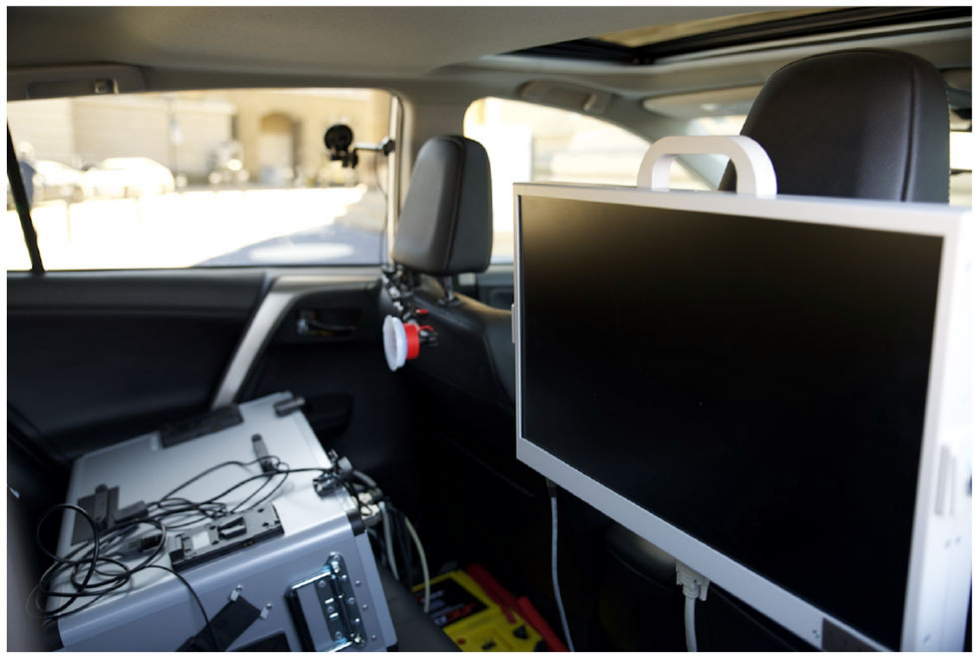
Data collection computer and monitor in the backseat of the instrumented vehicle.

The data from all devices was synced with vehicle data during data collection. A computer and monitor in the back seat allowed for real-time monitoring of data (Figure 3).

### 2.3. Parking Search Task

The task was designed to induce only the loads of visually searching for parking while driving rather than the task of parking itself. Participants were asked to identify legal, vacant parking spaces that were in their direction of travel and on the same street they were on. In the parking-search condition, there were four predetermined sections of each route where participants were asked to search for parking continuously (these sections ranged from approximately 400–800 m). They were told to verbally announce each space they encountered that they understood to be vacant and legal. After each announcement, they were told whether the parking space was indeed legal or why it was not. They then continued to search for parking. Participants were not asked to stop nor park. They were given turn-by-turn directions during the experiment (in both conditions) to eliminate the navigation component of the parking search; although not investigated in our experiment, this component is expected to further distress drivers in real-life scenarios.

### 2.4. Procedure

**Figure 4:**
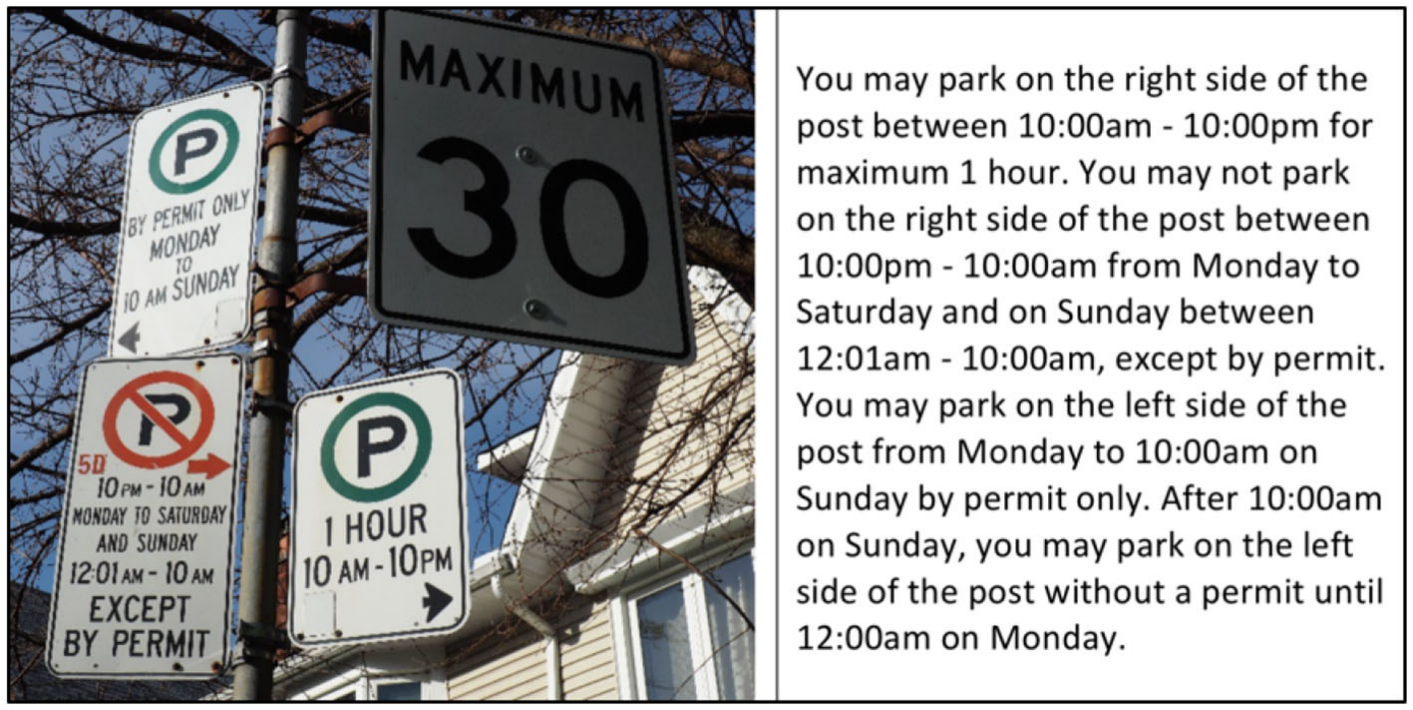
Example of sign explanation in parking instructions booklet provided to the participants.

The experiment contained both an off-road and an on-road component and took an average of 2 h. Participants first read and signed the informed consent form. They provided their driver's license to verify their age and license type and were aware that a scanned record was made for insurance purposes. After signing the informed consent document, participants filled out questionnaires to gain insights into their driving behavior and history. Participants were then given an instructional booklet that provided a brief overview of parking rules in Toronto and explained the restrictions described by parking signage found along the experimental driving routes (Figure 4). After going through the booklet on their own, participants were administered a five-question quiz and were assured that their performance on the quiz would not affect their participation in the rest of the experiment. The multiple-choice quiz tested their understanding of parking signage; after each question, if they chose an incorrect response, the investigator discussed the correct answer with the participants. The purpose of the booklet and quiz was to ensure that all participants had the same minimum level of exposure and understanding of the parking restrictions and signs in the area.

Participants were then taken to the instrumented vehicle and seated in the driver seat. They were given time to adjust the seat position and mirrors. The lead investigator sat in the passenger seat and a research assistant was seated behind the investigator, operating the data collection computer. Participants first completed a 5- to 10-min familiarity drive to allow them to get used to the vehicle. They were told that the investigator would provide them with turn-by-turn directions and that they should ask questions about operating the vehicle during the familiarity drive as talking during the experiment would be discouraged. The familiarity drive was on roads similar to the experimental routes. After the familiarity drive, the participants were outfitted with the physiological sensors and the head-mounted eye-tracking device, which was calibrated before each experimental route. They then completed the two experimental routes, one with the searching for parking task and the other serving as the baseline. As previously stated, the order of the routes and the conditions were fully counterbalanced across the participants to remove the potential route and order confounds. After each experimental route, participants completed the NASA Task Load Index (Hart and Staveland, 1988). As part of the questionnaire, they were required to complete a pairwise comparison of six types of workload based on which they felt contributed more to their workload. This calibration was done only once per participant after the first drive. For both drives, drivers rated the extent to which they felt six different types of demand (e.g., mental, physical) during the drive. After the second route, participants exchanged seats with the investigator and were driven back to campus and given their compensation.

## 3. Analysis

**Figure 5:**
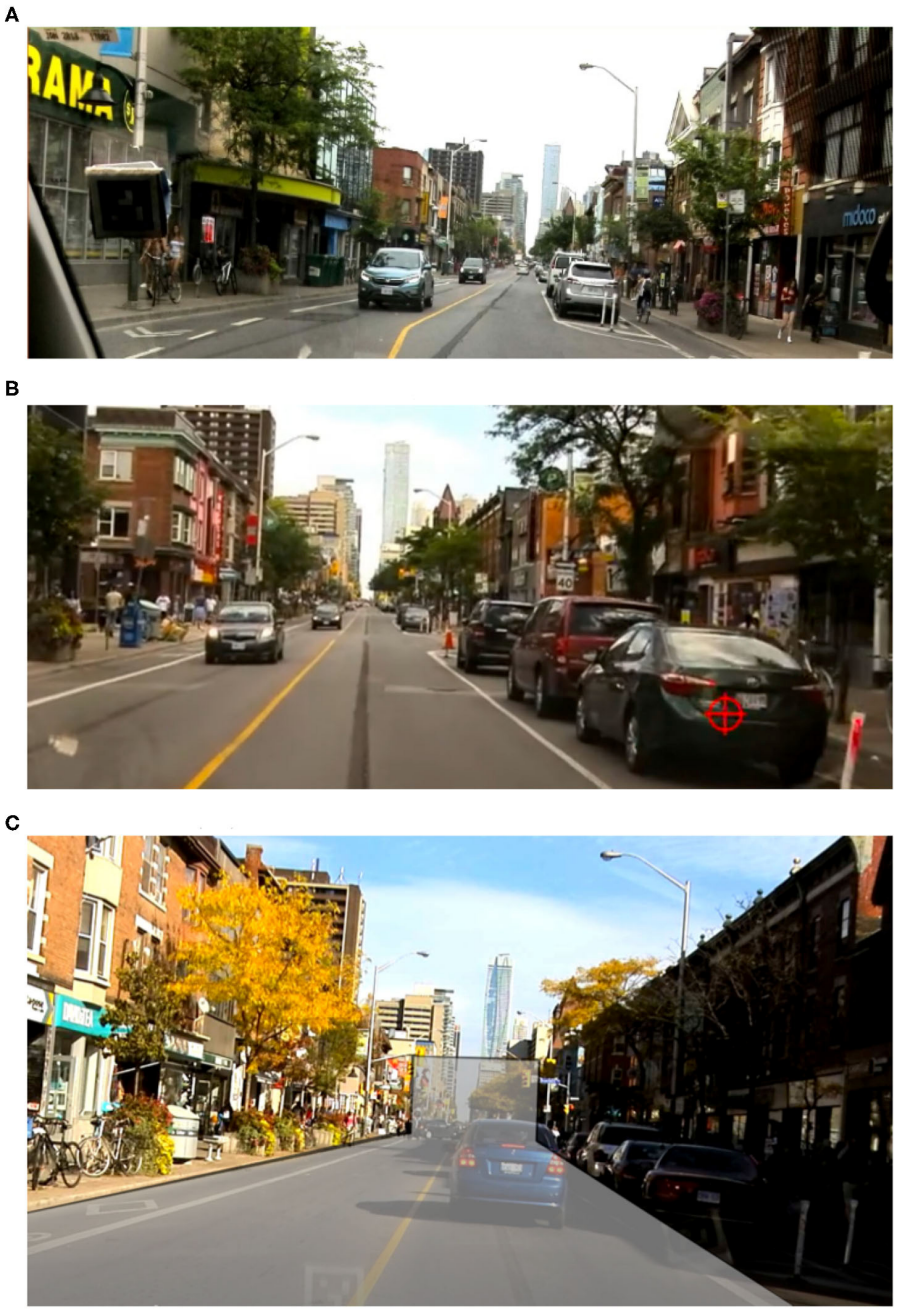
Views from the head-mounted camera on the Dikablis eye-tracker used for glance analysis. **(A)** Snapshot of Bloor St. **(B)** Gaze position indicated by the red cross-hair. **(C)** Bounded region considered “on-road”.

The experiment produced vehicle, physiological, eye-tracking, and subjective data from 28 participants each driving two 15- to 20-min routes under the parking-search and baseline conditions. While both routes were similar in length and complexity, they contained a variety of different streets and intersections. To mitigate these differences, each route included the same 540 m stretch of Bloor St. driven west to east by all participants, once under the baseline condition and once while searching for parking. Participants had already completed at least two sections of parking search before reaching the region, regardless of whether it was their first or second drive. For these reasons, this region was the focus of analysis for vehicle control, physiology, and glances. However, perceived workload was assessed at the end of each route, and therefore its analysis did not focus on the Bloor St. stretch. The Bloor St. stretch contains a single lane in each direction, each with a separated bike lane (Figure 5A). There is paid street parking allowed at parking bays indicated by pavement markings, signs, and bollards; parking on the right side of the street was observed to be almost fully-occupied at the times when the experiment took place (by reviewing videos post-experiment), with 3 or 4 spaces free out of 25 on average in the region.

### 3.1. Vehicle Control

We captured vehicle control through speed and lane position as well as their variability, measured as standard deviation (within participants) and all provided by the MobilEye device. Traffic flow, signal status, and pedestrian behavior could not be controlled during the experiment, thus there were many instances where participants were forced to stop or slow down, regardless of the speed they would normally choose. It was observed that on the Bloor St. stretch, speeds under 15 km/h were driven when participants were either slowing or stopping due to interruptions in the road, such as a red light, vehicles parking, pedestrians crossing, or congested traffic. Therefore, when calculating average speed and standard deviation of speed, only data recorded for speeds above 15 km/h was considered; drivers drove above 15 km/h 68.8% of the time (on average) when driving the Bloor St. stretch.

For all other vehicle measures, the entire set of data from the stretch was used. Lane position was recorded via the Distance to the Left Lane value provided by the MobilEye System (located 6 cm right of the center of the front windshield, Figure 1); this is calculated by the device using lane marking detection on video captured by its internal camera. Vehicle measures between the two experimental conditions were compared by paired *t*-tests. Time spent driving the Bloor St. stretch and time spent driving the stretch above 15 km/h were also compared between conditions using paired *t*-tests and applied as offset variables where appropriate in the statistical analysis of glance metrics.

### 3.2. Subjective Workload

The NASA TLX was administered after both experimental drives, with participants completing the pairwise comparison section only after the first drive. This pairwise comparison of six workload types produced a weighting for each participant, 5 being the type of workload they felt most contributed to their drive and 0 being the least. An average workload score (from 0 to 20) was calculated for each condition with these weights and the participant ratings (from 0 to 20) of the amount of each type of workload they experienced. The overall self-reported workload, and its components, were compared between the two routes via paired *t*-tests.

### 3.3. Physiological Measures

Physiological measures included average heart rate (calculated from EKG signals) and average galvanic skin conductance. Paired *t*-tests were carried out to compare the two experimental conditions with regard to physiological measures.

### 3.4. Glance Measures

Glances were coded by reviewing eye tracking videos (Figure 5B) and determining the periods when the driver had an off-road glance, i.e., was looking outside of the perimeter deemed on-road (Figure 5C). The length of a glance included both the fixation on the area of interest as well as the saccade to the area before the fixation, as defined by the International Organization for Standardization (ISO 15007-1). Glances less than 100 ms were removed from analysis as they may not represent meaningful fixations (Crundall and Underwood, 2011). In addition, because our interest was on searching for parking while driving, glances during periods where drivers were slowing to a stop, stopped (generally at a red traffic signal or to allow pedestrians to cross the street, not because they were searching for a parking spot), or following a very slow-moving vehicle were not of interest. Drivers tended to scan the environment far more during these periods, greatly skewing results. Therefore, glances were filtered to include only those made while the vehicle was moving above 15 km/h.

Based on an on-road study using an eye-tracker, it was reported that drivers rarely glance off the road for longer than 1.6 s (Sodhi et al., 2002); in addition, through a naturalistic driving study, it has been shown that glances off the forward roadway of over 2 s double the risk of a crash (Klauer et al., 2006). These two thresholds (1.6 and 2 s) are used widely in the study of driver distraction (e.g., Sodhi et al., 2002; Horrey and Wickens, 2007; Hallihan et al., 2011; Reimer et al., 2014). Thus, we also used these thresholds in our analysis. It should be noted that Klauer et al. (2006) utilized video recordings of the participants' face to assess gaze direction. Thus, their method is likely not as precise as our study's assessment of glance duration (hence the label “off the forward roadway” as opposed to “off-road”); however, the naturalistic nature of the study entails a high level of ecological validity.

Our glance measures included percentage of time looking away from the road, average off-road glance duration, rate of off-road glances per minute, and rate of shorter (<1.6 s) and long (>2 s) off-road glances per minute. Percent time and glance duration measures were analyzed with paired *t*-tests. Number of glances data were non-normal, thus were modeled through generalized linear models with the Poisson distribution and log link function, and with task condition (baseline or parking-search) as the predictor variable. The time spent above 15 km/h in minutes was used as an offset variable; therefore, the models predicted rate of glances (/min). Repeated measures were accounted for using generalized estimating equations.

## 4. Results

As mentioned earlier, some participants had to be dropped from analysis of some measures due to equipment malfunctions. Table 1 summarizes the number of participants whose data were analyzed for each measure, as well as their gender and age information.

**Table 1:** Number of participants with usable data for each measure.

**Measure**	***N***	**Age mean, standard deviation**
NASA TLX	14 male, 14 female	41.9, 5.7
Driving duration, speed	13 male, 13 female	42.2, 5.7
Lane position	12 male, 13 female	42.5, 5.7
Galvanic skin response	12 male, 12 female	42.4, 5.9
Heart rate	11 male, 12 female	42.6, 6.0
Glance measures	9 male, 7 female	42.8, 5.7

### 4.1. Vehicle Control

**Figure 6:**
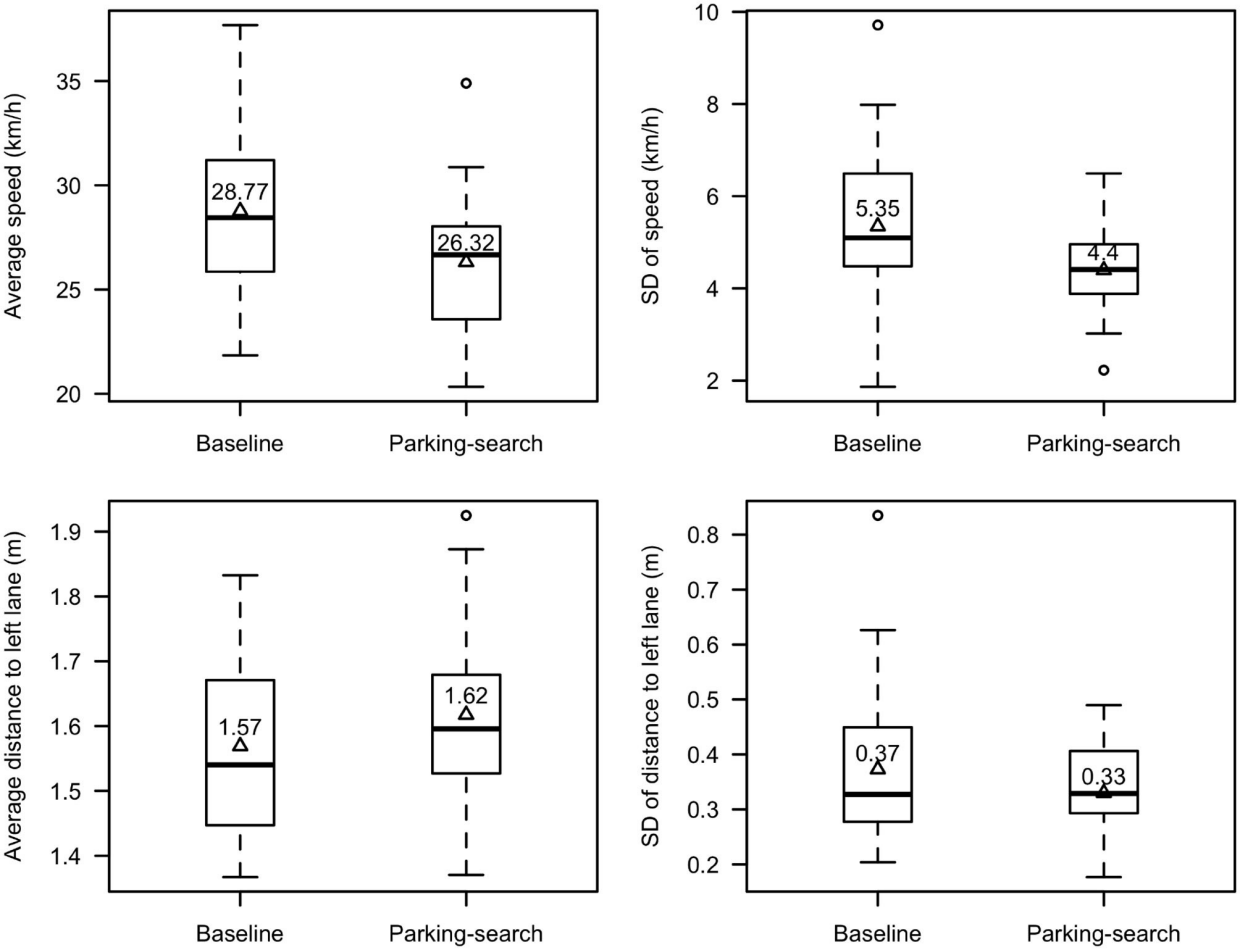
Average and standard deviation of speed and lane position. The mean is reported in the boxplot and indicated with a triangle.

The time spent driving the Bloor St. stretch was not expected to significantly differ between conditions, as the stretch contained 2 traffic signals and various traffic conditions that greatly affect this measure regardless of whether the searching task was performed. Indeed, there was no significant difference; it took participants an average of 77.2 s to complete the stretch while searching for parking (SD = 25.2 s) and 73.0 s in the baseline (SD = 17.1 s); *t*_(25)_ = 0.66, *p* = 0.51. When considering only speeds above 15 km/h (Figure 6), the average speed was found to be significantly higher in the baseline condition (M = 28.8 km/h, SD = 4.0 km/h) than when drivers were searching for parking (M = 26.3 km/h, SD = 3.2 km/h), *t*_(25)_ = 2.34, *p* = 0.03, Cohen's d = 0.68. The standard deviation of speed above 15 km/h was also, on average, significantly higher in the baseline (M = 5.5 km/h, SD = 1.8 km/h) than in the parking-search condition (M = 4.4 km/h, SD = 1.0 km/h), *t*_(25)_ = 2.27, *p* = 0.03, Cohen's d = 0.67. The average distance to the left lane significantly differed between conditions (Figure 6); drivers drove further from the left lane when searching for parking (M = 1.62 m, SD = 0.13 m) than in the baseline (M = 1.57 m, SD = 0.13 m), *t*_(24)_ = 2.11, *p* = 0.045, Cohen's d = 0.37. The standard deviation of distance to the left lane did not differ significantly when participants searched for parking (M = 0.33 m, SD = 0.09 m) compared to their baseline (M = 0.37 m, SD = 0.14 m), *t*_(24)_ = 1.54, *p* = 0.14.

### 4.2. Subjective Workload

**Figure 7:**
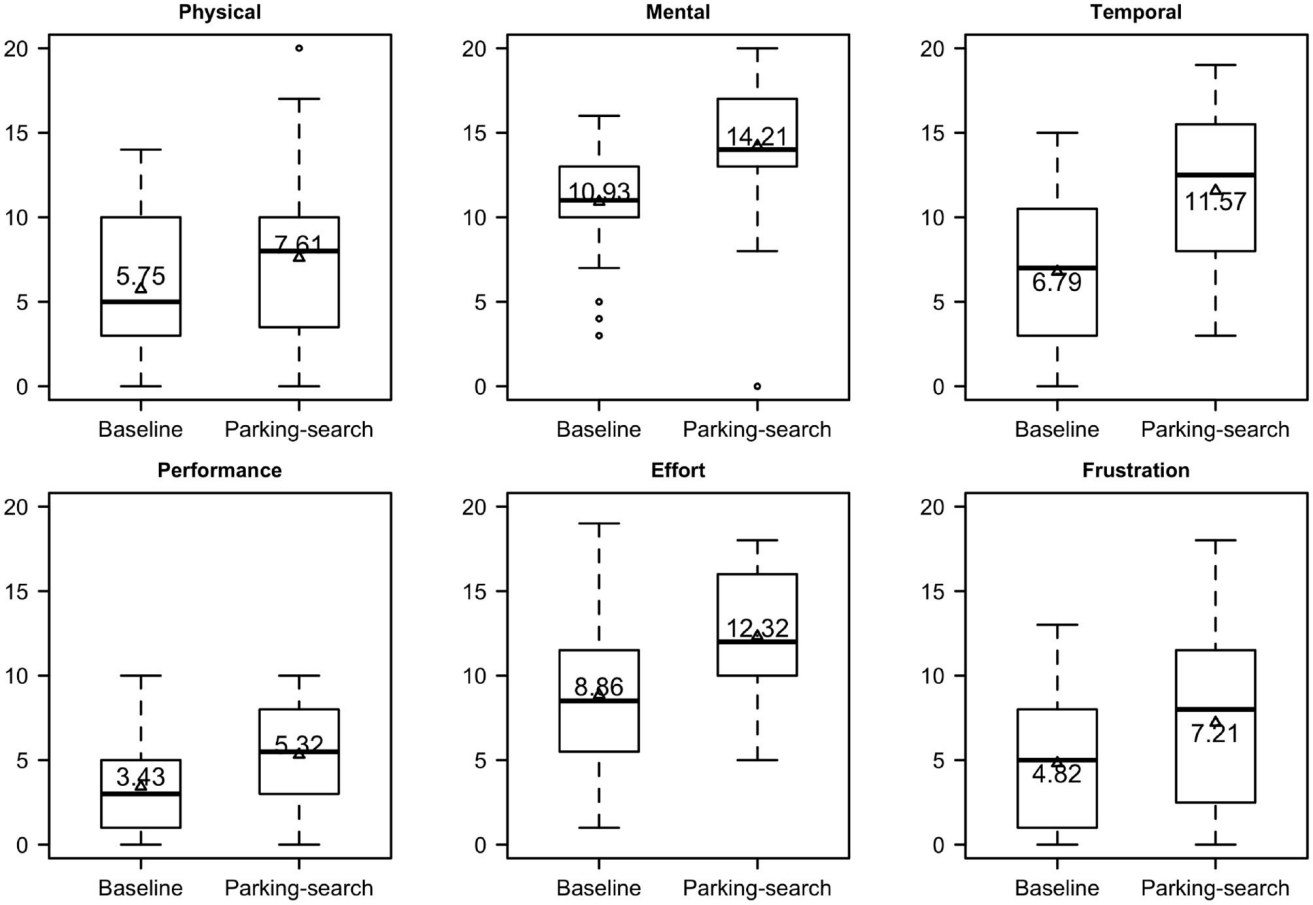
NASA TLX un-weighted ratings for each workload category and task condition.

The overall NASA TLX score was significantly higher during the parking-search route (M = 54.63, SD = 14.53) than in the baseline route (M = 39.02, SD = 11.27), *t*_(27)_ = 5.36, *p* < 0.001, Cohen's d = 1.19. Figure 7 displays the raw (un-adjusted by weighting, for comparison) rating for each specific type of workload by task conditions. These box plots as well as the ones presented later depict the minimum, maximum, 1st and 3rd quartiles, and the median, as well as the mean overlaid on the box as triangles along with its value. The difference between parking-search and baseline routes for the individual workload components were all significant: physical [1.86; *t*_(27)_ = 2.6, *p* = 0.02, Cohen's d = 0.43], mental [3.28; *t*_(26)_ = 3.7, *p* = 0.001, Cohen's d = 0.84], temporal [4.78; *t*_(27)_ = 5.5, *p* < 0.001, Cohen's d = 1.03], performance [1.9; *t*_(27)_ = 4.4, *p* < 0.001, Cohen's d = 0.69], effort [3.46; *t*_(27)_ = 3.4, *p* = 0.002, Cohen's d = 0.87] and frustration [2.40; *t*_(27)_ = 2.5, *p* = 0.02, Cohen's d = 0.51].

### 4.3. Physiological Measures

The average skin conductance did not differ significantly between conditions, *t*_(23)_ = 1.51, *p* = 0.14. The difference in average heart rate approached significance, with participants exhibiting a potential increase when searching for parking (M = 78.99 beats/minute (bpm), SD = 12.34 bpm) over the baseline (M = 77.89 bpm, SD = 12.55 bpm), *t*_(22)_ = 1.76, *p* = 0.09.

### 4.4. Glance Measures

**Figure 8:**
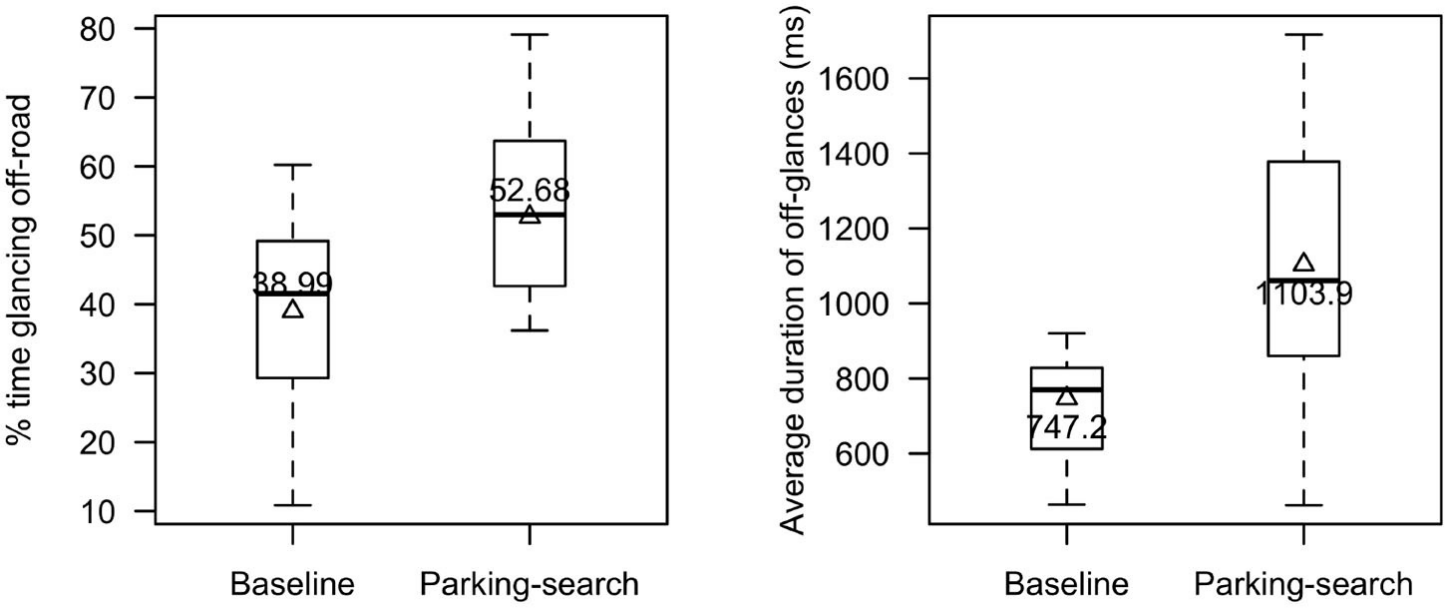
Percent time glancing off-road and average duration of off-road glances.

**Figure 9:**
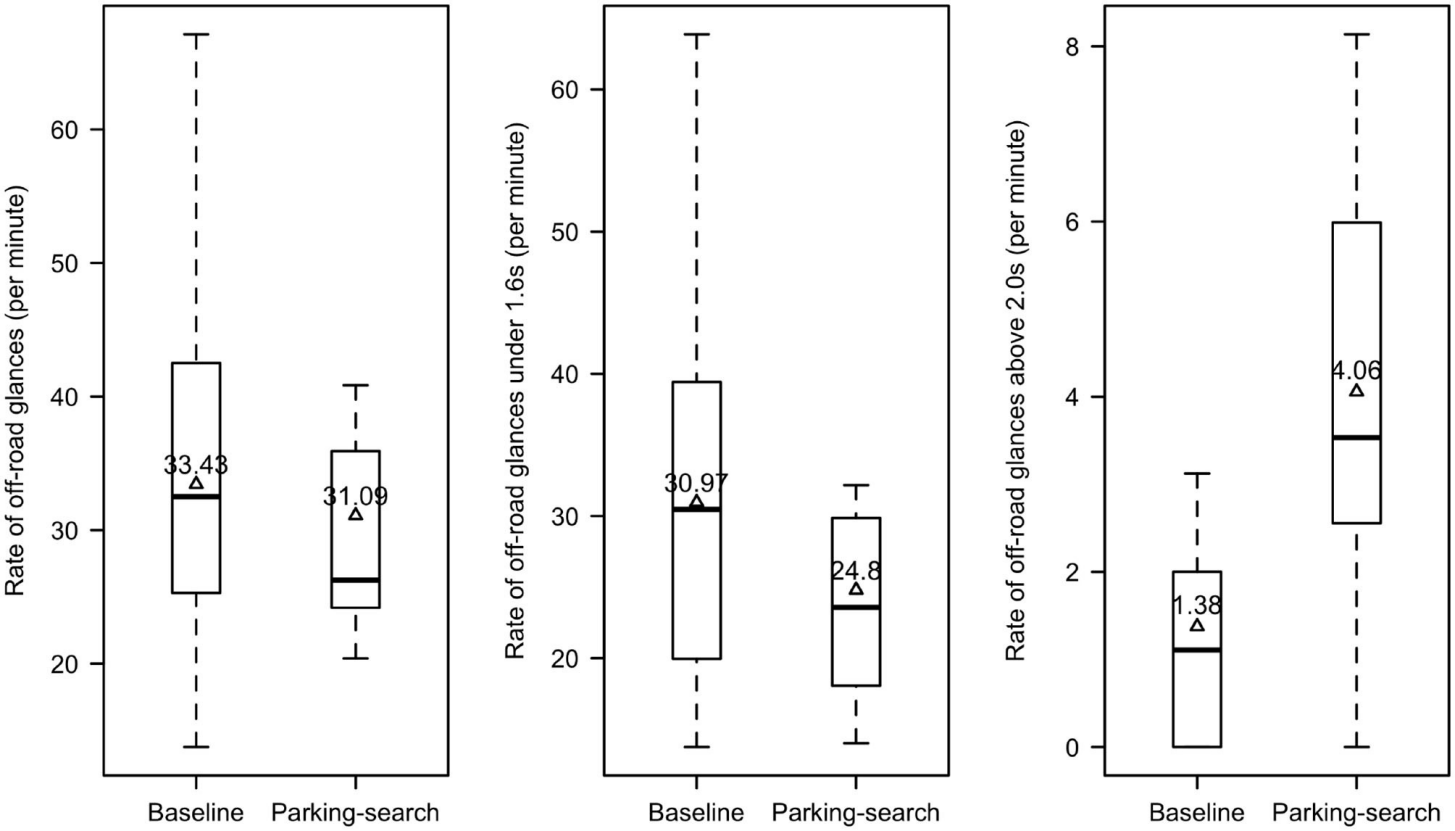
Rates of off-road glances (total, under 1.6 s, above 2 s) per minute.

As shown in Figure 8, participants spent more time looking off-road (driving over 15km/h) when searching for parking (M = 53%, SD = 17%) than in the baseline condition (M = 39%, SD = 15%), *t*_(15)_ = 2.80, *p* = 0.01, Cohen's d = 0.70. Further, considering only when driving over 15 km/h, participants had longer off-road glances while searching for parking (M = 1.1 s, SD = 0.4 s) than in the baseline (M = 0.7 s, SD = 0.2 s); *t*_(15)_ = 3.5, *p* = 0.003, Cohen's d = 0.87. Rate of all off-road glances was not significant, χ^2^(1) = 1.36, *p* = 0.24; however, rate of off-road glances under 1.6 s, χ^2^(1) = 10.94, *p* < 0.001, and the rate of off-road glances over 2 s, χ^2^(1) = 22.11, *p* < 0.001, were significant. The rate of off-road glances under 1.6 s was 28% higher in the baseline condition compared to the parking-search condition, 95% CI = (9, 47%), whereas the rate of off-road glances over 2 s was 235% higher in the parking-search condition than in the baseline, 95% CI = (81, 520%). The plots for significant findings in glance rates are found in Figure 9.

## 5. Discussion

This is the first known study that attempts to quantify the effect searching for parking has on drivers through an on-road experiment. We aimed this study to act as a first step into understanding how the necessity for searching for street parking affects the safety of the road environment. The use of an instrumented vehicle and head-mounted eye tracker allowed for relatively precise data collection in a real-world environment, compared to simulator and naturalistic studies. The parking-search task designed for this experiment was a simplified version of the search for parking drivers normally experience. Participants were not required to navigate, did not have any time pressures enforced on them, and were only required to search for parking that was in the same direction and on the same street as they were going. We found evidence that searching for parking has a measurable effect on drivers, particularly on their perceived workload, vehicle speed and lane position and glance behavior. Drivers reported an increase in workload and were found to drive slower and closer to the curb when searching for parking. They also exhibited longer off-road glances and more frequent long off-road glances. Given the simplification of the parking-search task in this experiment, it is expected that drivers in a similarly complex environment are affected even more so in natural conditions.

Under the condition of searching for parking in which they experience increased perceived workload, participants drove slower on average. Lowering speed is often seen as a compensatory strategy that has been observed when drivers experience high visual workload (Engström et al., 2005). A decrease in the standard deviation of average speed was also observed, however the lower speed variability may be a statistical artifact of the generally lower speeds exhibited when searching for parking. The Edquist et al. (2012) simulator study found that drivers drove further from the curb in similar conditions to the Bloor St. stretch (i.e., urban environment, single lane in each direction, fully occupied parking bays along both sides) analyzed in our study; it was suggested that this may be dangerous as vehicles were positioned closer to oncoming traffic. Worth noting is that the influence of this potential danger is likely minimized when driving in a simulator study, possibly leading to riskier behavior than found in a real environment. Our analysis revealed that when tasked with searching for parking in similarly visually complex conditions, participants drove closer to the curb. Drivers may have purposefully kept themselves farther from oncoming traffic while they engaged in a potentially distracting task. However, it is also possible that they drifted nearer to the parking bays as they visually inspected them for vacancy, or to better read parking signs with small letter sizing. Interestingly, it was observed that participants received many of their cues regarding the legality of parking spaces by the presence of other parked cars, rather than by reading the parking signage thoroughly. For this reason, it was difficult to investigate how their understanding of the parking rules may have affected the task, and modifications to the experimental methodology would be needed to do so. In addition, further research is needed to comment on the role reading signs plays in the search for parking. There was no increase in lane keeping variability observed, despite the hypothesis that it would increase under heightened visual load as reported in another study (Engström et al., 2005). The lack of significance in our study may be due to the generally low speeds of the driving area which allowed participants to maintain their course with minimal deviation; it is suggested that further research be done in an area where speeds average above 40 km/h. A lack of statistical power may also explain these and other non-significant findings.

Participants self-reported a clear increase in workload between driving under the baseline condition and driving when periodically searching for parking. While not explicitly studied, we found evidence that time pressure could be induced by the social aspects of driving (i.e., slowing traffic) and not only by the driver's own motivation to complete the task as quickly as possible. The results of the NASA TLX questionnaire revealed that the largest average difference in demand reported was in temporal demand; this indicates that drivers did feel time pressure when searching for parking even though there was no deadline to reach a destination. It seems then that the rate at which they performed the task was at least partially imposed on them by external pressures, social or otherwise.

**Figure 10:**
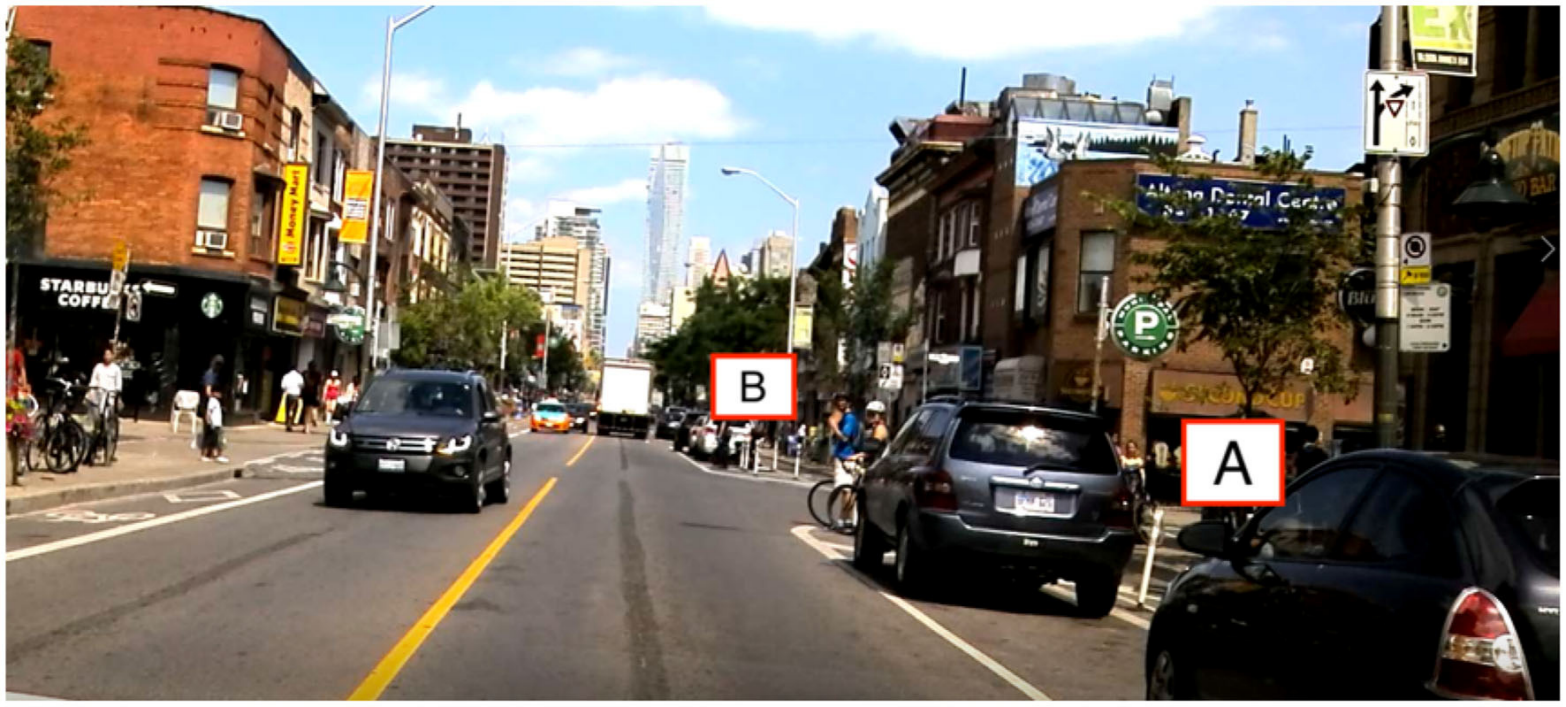
Fixation on point B allows more of the road ahead to be maintained in the driver's central vision than fixation on point A.

This study revealed that searching for parking brought on measurable differences in glance behavior. Participants exhibited fewer off-road glances under 1.6 s but more glances over 1.6 s (as suggested by the minimal change in overall rate of glances) when searching for parking compared to the baseline. This suggests an adjustment of visual scanning behavior when searching for parking by lessening the number of short glances they perform to allow more long off-road glances. Long off-road glances can reduce the driver's ability to respond to unexpected events on the road (Liang et al., 2012). Drivers exhibiting more frequent long off-road glances may contribute to a more dangerous driving environment. Even though participants were found to decrease the number of short off-road glances when searching for parking, the total percentage of off-road glance duration was still higher in this condition compared to the baseline. It is important to note that not all off-road glances are equivalent. Glances made off-road far ahead of the vehicle (Figure 10, point B) still allow the driver to maintain the environment ahead in their field of view, while fixations on points closer to the vehicle with a higher angular velocity (Figure 10, point A) have a reduced portion of the road ahead held in their view. Further analysis on the angular velocity of fixation points is needed to determine how unsafe long off-road glances are. In addition, some off-road glances are necessary to ensure a safe environment, such as glances to a pedestrian about to cross the street.

Physiological signals (heart rate and galvanic skin response, GSR) were expected to reflect an increase in workload. Although heart rate variability is another measure of workload, it was not analyzed given that our study did not provide the recommended 5-min minimum of baseline signal to properly assess any change in HRV between task conditions (Shaffer and Ginsberg, 2017). However, average heart rate showed only a slight increase approaching significance when drivers were searching for parking, and average GSR did not show any significant difference between task conditions. The lack of significance may again be due to a lack of power resulting from our limited sample size or from the variability introduced from the driving environment encompassing uncontrolled factors (e.g., pedestrian jay-walking, traffic signal status, behavior of other traffic) that may have impacted the driver's physiological state more than the searching for parking task. Another possible factor, given that participants did self-report a clear increase in workload, is that increased sensory information taken in when searching for parking caused a decrease in heart rate counter to the rise experienced due to stress. This phenomenon, known as “*sensory intake*,” has been suggested to occur when drivers are intently focused on absorbing sensory information (e.g. visually searching for an open parking space) (Mehler et al., 2008). The GSR sensors used in the study could also be unreliable due to the noise in the signal brought on by the vibrations in the vehicle and the movement of the participant; a more robust placement than the bottom of the foot may have achieved better results.

Our work adds to the growing body of on-road experimental studies that aim to quantify driver behaviors in real-world environments. We found that, when searching for parking, drivers exhibited some compensatory behaviors which are conducive to a safer driving environment, such as reduced speed. They also exhibited behaviors which can be considered unsafe, such as increased off-road glances over 2 s. It is recognized that, though statistically significant, the differences in speed and lane position between task conditions are relatively small. Further investigation in different types of road environments is needed to conclude whether such differences can contribute to the heightened crash risk that has been exhibited in areas that allow street parking. This work serves as an important initial step in investigating how searching for parking affects drivers, and acts as an invitation to continue researching a common, often taxing task for drivers that may contribute to crash risk in busy urban areas. Such findings would justify the development of measures, such as changes in road design or parking search assistance via mobile applications, that provide additional benefits to drivers beyond reducing traffic congestion and parking payment efficiency.

## Data Availability Statement

The datasets presented in this article are not readily available because the raw data contains confidential information as defined in the agreement with the University of Toronto Research Ethics Board. Requests to access the datasets should be directed to Birsen Donmez.

## Ethics Statement

The studies involving human participants were reviewed and approved by University of Toronto Research Ethics Board. The patients/participants provided their written informed consent to participate in this study. Written informed consent was obtained from the individual(s) for the publication of any potentially identifiable images or data included in this article.

## Author Contributions

CP was the graduate student who designed this experiment, collected, and analyzed the data under the supervision of and with feedback from BD. CP drafted this manuscript with feedback from and revisions by BD. All authors contributed to the article and approved the submitted version.

## Conflict of Interest

The authors declare that the research was conducted in the absence of any commercial or financial relationships that could be construed as a potential conflict of interest.

## References

[B1] Avcı C. Akbaş A. Yüksel Y. (2014). “Evaluation of statistical metrics by using physiological data to identify the stress level of drivers,” in Proceedings of the 3rd International Conference on Environment, Chemistry and Biology IPCBEE, Vol. 78 (Singapore: IACSIT Press), 124–128. 10.7763/IPCBEE

[B2] Box P. C. Levinson H. S. (2004). Curb-parking problems: overview. J. Transport. Eng. 130, 1–5. 10.1061/(ASCE)0733-947X(2004)130:1(1)

[B3] Brooke S. Ison S. Quddus M. (2014). On-street parking search. Transport. Res. Rec. J. Transport. Res. Board 2469, 65–75. 10.3141/2469-08

[B4] Cao Y. Yang Z. Z. Zuo Z. Y. (2017). The effect of curb parking on road capacity and traffic safety. Eur. Transport Res. Rev. 9:4 10.1007/s12544-016-0219-3

[B5] Cooper P. J. (1990). Differences in accident characteristics among elderly drivers and between elderly and middle-aged drivers. Accident Anal. Prevent. 22, 499–508. 10.1016/0001-4575(90)90044-L 2222712

[B6] Crundall D. Underwood G. (2011). “Visual attention while driving: measures of eye movements used in driving research,” in Handbook of Traffic Psychology, ed B. E. Porter (San Diego, CA: Academic Press), 137–148. 10.1016/B978-0-12-381984-0.10011-6

[B7] Daisa J. M. Peers J. B. (1997). “Narrow residential streets: do they really slow down speeds?” in Proceedings of the Institute of Transportation Engineers 67th Annual Meeting (Tampa, FL: Institute of Transportation Engineers).

[B8] Dingus T. McGehee D. Hulse M. Jahns S. Manakkal N. (1995). Travtrek Evaluation Task C3–Camera Car Study. Technical report, Turner-Fairbank Highway Research Centre, McLean, VA.

[B9] Dingus T. A. Guo F. Lee S. Antin J. F. Perez M. Buchanan-King M.. (2016). Driver crash risk factors and prevalence evaluation using naturalistic driving data. Proc. Natl. Acad. Sci. U.S.A. 113, 2636–2641. 10.1073/pnas.1513271113 26903657 PMC4790996

[B10] Edquist J. Rudin-Brown C. M. Lenné M. G. (2012). The effects of on-street parking and road environment visual complexity on travel speed and reaction time. Accident Anal. Prevent. 45, 759–765. 10.1016/j.aap.2011.10.001 22269567

[B11] Engström J. Johansson E. Östlund J. (2005). Effects of visual and cognitive load in real and simulated motorway driving. Transport. Res. F Traffic Psychol. Behav. 8, 97–120. 10.1016/j.trf.2005.04.012

[B12] Greibe P. (2003). Accident prediction models for urban roads. Accident Anal. Prevent. 35, 273–285. 10.1016/S0001-4575(02)00005-2 12504148

[B13] Hallihan G. Mayer A. Caird J. Milloy S. (2011). Effects of hybrid interface on ecodriving and driver distraction. Transport. Res. Rec. J. Transport. Res. Board 2248, 74–80. 10.3141/2248-10

[B14] Hampshire R. Shoup D. (2018). What share of traffic is cruising for parking? J. Transport Econ. Policy 52, 184–201.

[B15] Hart S. G. (2006). “NASA-Task Load Index (NASA-TLX); 20 years later. Proc. Hum. Fact. Ergon. Soc. Annu. Meet. 50, 904–908. 10.1177/154193120605000909

[B16] Hart S. G. Staveland L. E. (1988). Development of NASA-TLX (task load index): results of empirical and theoretical research. Adv. Psychol. 52, 139–183. 10.1016/S0166-4115(08)62386-9

[B17] Healey J. Picard R. (2005). Detecting stress during real-world driving tasks using physiological sensors. IEEE Trans. Intell. Transport. Syst. 6, 156–166. 10.1109/TITS.2005.848368

[B18] Hennessy D. A. Wiesenthal D. L. (1999). Traffic congestion, driver stress, and driver aggression. Aggress. Behav. 25, 409–423. 10.1002/(SICI)1098-2337(1999)25:6<409::AID-AB2>3.0.CO;2-0

[B19] Highway Research Board (1971). Parking Principles. Technical report, National Academy of Sciences, Washington, DC.

[B20] Horrey W. Wickens C. (2007). In-vehicle glance duration: distributions, tails, and model of crash risk. Transport. Res. Rec. J. Transport. Res. Board 2018, 22–28. 10.3141/2018-04

[B21] Kaptein N. Theeuwes J. Van Der Horst R. (1996). Driving simulator validity: some considerations. Transport. Res. Rec. J. Transport. Res. Board 1550, 30–36. 10.1177/0361198196155000105

[B22] Klauer S. Dingus T. Neale V. Sudweeks J. Ramsey D. (2006). The Impact of Driver Inattention On Near Crash/Crash Risk: An Analysis Using the 100-Car Naturalistic Driving Study Data. Technical report. Virginia Tech Transportation Institute 10.1037/e729262011-001

[B23] Lerner-lam E. Celniker S. R. Halbert G. W. Chellman C. Ryan S. (1992). Neo-traditional neighborhood design and its implications for traffic engineering. Instit. Transport. Eng. J. 62, 17–25.

[B24] Liang Y. Lee J. D. Yekhshatyan L. (2012). How dangerous is looking away from the road? Algorithms predict crash risk from glance patterns in naturalistic driving. Hum. Fact. 54, 1104–1116. 10.1177/0018720812446965 23397818

[B25] Marshall W. Garrick N. Hansen G. (2008). Reassessing on-street parking. Transport. Res. Rec. J. Transport. Res. Board 2046, 45–52. 10.3141/2046-06

[B26] McGwin G. Jr. Brown D. B. (1999). Characteristics of traffic crashes among young, middle-aged, and older drivers. Accident Anal. Prevent. 31, 181–198. 10.1016/S0001-4575(98)00061-X 10196595

[B27] Mehler B. Reimer B. Coughlin J. F. (2012). Sensitivity of physiological measures for detecting systematic variations in cognitive demand from a working memory task: an on-road study across three age groups. Hum. Fact. 54, 396–412. 10.1177/0018720812442086 22768642

[B28] Mehler B. Reimer B. Pohlmeyer A. E. Coughlin J. F. (2008). The association between heart rate reactivity and driving performance under dual task demands in late middle age drivers. Adv. Transport. Stud. 53–70. 10.4399/97888548259876

[B29] Ponnambalam C. T. Cheng R. Donmez B. (2018). Effects of searching for street parking on driver behaviour and physiology: results from an on-road instrumented vehicle study. Proc. Hum. Fact. Ergon. Soc. Annu. Meet. 62, 1404–1408. 10.1177/1541931218621320

[B30] Regan M. A. Lee J. D. Young K. L. (2008). Driver Distraction: Theory, Effects, and Mitigation. Boca Raton, FL: CRC Press; Taylor & Francis Group 10.1201/9781420007497

[B31] Reimer B. Mehler B. Coughlin J. F. (2016). Reductions in self-reported stress and anticipatory heart rate with the use of a semi-automated parallel parking system. Appl. Ergon. 52, 120–127. 10.1016/j.apergo.2015.07.008 26360202

[B32] Reimer B. Mehler B. Donmez B. (2014). A study of young adults examining phone dialing while driving using a touchscreen vs. a button style flip-phone. Transport. Res. F Traffic Psychol. Behav. 23, 57–68. 10.1016/j.trf.2013.12.017

[B33] Shaffer F. Ginsberg J. P. (2017). An overview of heart rate variability metrics and norms. Front. Public Health 5:258. 10.3389/fpubh.2017.00258 29034226 PMC5624990

[B34] Sisiopiku V. P. (2001). “On-street parking on state roads,” in Proceedings of ITE 2001 Annual Meeting and Exhibit (Chicago, IL).

[B35] Sodhi M. Reimer B. Llamazares I. (2002). Glance analysis of driver eye movements to evaluate distraction. Behav. Res. Methods Instrum. Comput. 34, 529–538. 10.3758/BF03195482 12564557

[B36] Weinberger R. Millard-Ball A. (2017). “Parking search caused congestion : where's all the fuss?” in Transportation Research Board 96th Annual Meeting (Washington, DC).

[B37] Zhang H. Smith M. R. H. Witt G. J. (2006). Identification of real-time diagnostic measures of visual distraction with an automatic eye-tracking system. Hum. Fact. J. Hum. Fact. Ergon. Soc. 48, 805–821. 10.1518/001872006779166307 17240726

